# Regional Time-Series Coding Network and Multi-View Image Generation Network for Short-Time Gait Recognition

**DOI:** 10.3390/e25060837

**Published:** 2023-05-23

**Authors:** Wenhao Sun, Guangda Lu, Zhuangzhuang Zhao, Tinghang Guo, Zhuanping Qin, Yu Han

**Affiliations:** 1 School of Automation and Electrical Engineering, Tianjin University of Technology and Education, Tianjin 300222, China; whsun@tute.edu.cn (W.S.);; 2 Tianjin Key Laboratory of Information Sensing & Intelligent Control, Tianjin 300222, China

**Keywords:** short-time gait recognition, feature fusion, time-series feature extraction, image generation networks

## Abstract

Gait recognition is one of the important research directions of biometric authentication technology. However, in practical applications, the original gait data is often short, and a long and complete gait video is required for successful recognition. Also, the gait images from different views have a great influence on the recognition effect. To address the above problems, we designed a gait data generation network for expanding the cross-view image data required for gait recognition, which provides sufficient data input for feature extraction branching with gait silhouette as the criterion. In addition, we propose a gait motion feature extraction network based on regional time-series coding. By independently time-series coding the joint motion data within different regions of the body, and then combining the time-series data features of each region with secondary coding, we obtain the unique motion relationships between regions of the body. Finally, bilinear matrix decomposition pooling is used to fuse spatial silhouette features and motion time-series features to obtain complete gait recognition under shorter time-length video input. We use the OUMVLP-Pose and CASIA-B datasets to validate the silhouette image branching and motion time-series branching, respectively, and employ evaluation metrics such as IS entropy value and Rank-1 accuracy to demonstrate the effectiveness of our design network. Finally, we also collect gait-motion data in the real world and test them in a complete two-branch fusion network. The experimental results show that the network we designed can effectively extract the time-series features of human motion and achieve the expansion of multi-view gait data. The real-world tests also prove that our designed method has good results and feasibility in the problem of gait recognition with short-time video as input data.

## 1. Introduction

Gait recognition refers to the technology that identifies a person by analyzing his or her gait information. In the past decades, gait recognition technology has been widely used in various fields, including human identification, motion analysis, disease diagnosis, and human-computer interaction [1,2,3]. As a new biometric feature recognition technology with potential, gait recognition has the advantages of being recognizable from a distance, easy to acquire, requiring low image quality, and not easy to hide. With the rapid development of computer vision technology, public security systems and intelligent video analysis systems combined with gait recognition have a wide technical demand in safeguarding public safety and improving the scientific management of smart cities [4,5,6].

Currently, there are two main approaches to gait recognition technology: sensor-based approaches and video-based approaches. With the development of sensor technology, gait recognition technology has also made significant progress. Sensor-based approaches [7,8,9] use multiple sensors, such as accelerometers, gyroscopes, and pressure sensors, to capture a variety of information about the human body, such as body posture, acceleration, angular velocity, and pressure distribution, which can provide rich recognition features for gait recognition. The method using sensor detection has better robustness and can be used both indoors and outdoors. However, the sensor-based approach requires high accuracy of the sensor and is susceptible to external interference. At the same time, wearing the sensors can easily subjectively affect a human subject’s movement habits, which leads to a larger error in recognition. In the security field, gait features can often only be obtained from short video information, so the method of wearing sensors also has strong limitations.

Video-based methods, on the other hand, obtain gait features from video data. Early methods used background subtraction to extract the main human silhouettes [10,11] and model the structure and transition process of gait silhouettes, including the gait energy image (GEI), frame difference entropy image, etc. The GEI and frame difference entropy images are used to represent the spatio-temporal series motion process of walking by combining the walking process of the detected object in the form of silhouette extraction into a new image. GEI is widely used in model-free gait recognition work. The advantage of this type of method is that the processing is relatively simple, using only traditional image processing methods to remove information such as background and human texture and focus on gait information. However, the recognition effect of this method depends on the completeness and continuity of the image, and it can easily lead to the loss of time-series information or misalignment during the modeling process, making the recognition accuracy much lower.

In recent years, with the development of hardware computing power and neural network research, the problems that can be solved using deep learning have become more extensive and numerous. These include the use of deep learning for more accurate image classification [12], biometric techniques in more scenarios [13,14], sequence data processing [15], etc. Similarly, research related to gait recognition using deep learning methods has become the mainstream approach in the field of gait recognition today. One of these methods is GaitSet, a depth set based gait recognition method proposed by Chao et al. [16]. Firstly, spatial features are extracted from the original gait silhouette using a convolutional neural network, and then the spatial features are compressed and integrated in the timeseries dimension. The GaitSet algorithm proposes a new view of treating gait as a collection containing independent frames, without requiring the order of the frames or even integrating video frames from different scenes. Most of the previous research works have used the whole gait data of the human body as network input for feature extraction. In contrast, GaitPart, proposed by Fan et al. [17], represents each part of the human body as an independent spatio-temporal series relationship. The highlight of GaitPart is that it focuses on the connections and differences in the shape of different parts of the human body while walking. This method of identifying gait through local modeling is easier to verify quantitatively. Some researchers have achieved gait recognition by studying the distribution patterns of position changes of body skeletal points [18,19,20]. For example, using Microsoft’s Kinect, a video stream with the distribution of human skeletal points is output directly from the original video stream. Each joint of the body in the video stream is represented as a point in 3D space. Later, static data such as limb length and dynamic data such as limb movement patterns are analyzed. However, gait recognition is susceptible to various interference factors such as dress, the carrying of objects or backpacks, and multiple views, among which changes in views have the most obvious impact on recognition performance. In practical applications, it is quite difficult to capture long-time continuous and complete gait data under multiple views. Therefore, cross-view gait recognition is an important challenge. In addition to the approach of using convolutional neural networks for uniform feature extraction of data from all views, some researchers have also adopted the approach of introducing a generative adversarial network (GAN) [21,22] to model the distribution of multi-view data. A gait generative adversarial network (GaitGAN) proposed by Yu et al. [23] normalizes the gait data from different views into gait data from lateral views. The method of converting multiple views into standard views by means of neural network learning before recognition has been proven to be effective. However, the details of the image cannot be expressed completely due to the lack of modeling the global relationship during the view conversion process. Moreover, as the span of the views increases, the error of the standard views obtained from the conversion becomes larger.

The above analysis suggests that to achieve more accurate and reliable gait recognition, it is most important to obtain gait data with complete time-series information and a sufficient amount of data. In order to achieve gait recognition under shorter input duration, a two-branch fusion gait recognition algorithm combining time-series data and silhouette information is proposed in this paper. The time-series information is modeled using a region coding network based on Transformer [24]. The expansion and integration of the silhouette data is implemented using a generative adversarial network with an added attention mechanism. In order to make full use of the feature information of the two-branch network, a feature fusion module is designed in this paper for the two dimensions of time series and contour, which differ significantly.

In summary, the contributions of this paper can be summarized as the following four points:

- A Transformer-based regional time-series coding network is designed. The joint position change information within and between each human region delineated in this paper is modeled, and effective time series features are extracted.
- A GAN-based gait data expansion network is designed. Only short-duration gait video data are input, and the gait silhouette data under multiple views are obtained by continuous training of the generator and discriminator to further expand the existing gait dataset.
- A feature fusion module based on bilinear matrix decomposition pooling is designed. The discrepancy between gait time-series features and contour features is effectively solved, and the data of both features are efficiently fused.
- The time-series coding network and data expansion network are tested on the OUMVLP-Pose and CASIA-B datasets, respectively, to verify the effectiveness of the algorithm. Meanwhile, the algorithm is validated in this paper using gait data collected in real scenes. The results show the effectiveness of the algorithm in this paper.

## 2. Methods

### 2.1. Overall Structure

**Figure 1:**
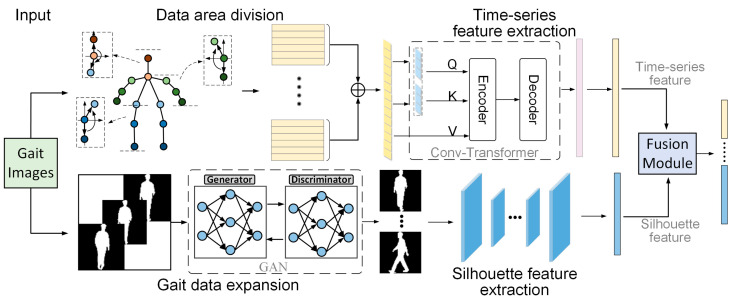
The overall structure of the algorithm in this paper.

The overall structure of the algorithm in this paper is shown in Figure 1. The input data is the frame sequence of the original RGB video, the keypoint location sequence data is obtained by the keypoint recognition algorithm, and the human silhouette image sequence is obtained by the background segmentation algorithm. This paper adopts a two-branch network structure. The timeseries data branch is a Transformer-based regional time series coding network. In this branch, the human body is divided into multiple regions according to the joint connection relationship. The relationships within and between regions are modeled by the timeseries coding network to characterize the unique positional relationships between limbs when a person walks. The silhouette data branch is expanded with a generative adversarial network incorporating an attention mechanism for gait silhouette data, followed by feature extraction using multilayer convolution. The feature vectors output from the timeseries data branch and the contour data branch are computationally fused by the feature fusion module to obtain the final gait feature data. In the following sections, the above method and network structure are described in detail.

### 2.2. Transformer-Based Regional Time Series Coding Network

In the process of extracting timeseries features for human joint position changes, a Transformer-based regional timeseries coding network is designed in this paper. For time-series data, Transformer is able to model global dependencies well. The basic Transformer consists of an encoder and a decoder. The encoder includes multiple Multi-Head Self-Attention modules and a position feedforward network (FFN), and the decoder is a cross-attention model inserted between the Multi-Head Self-Attention modules and the position feedforward network. As opposed to recurrent neural networks such as LSTM [25], Transformer models sequence information by embedding position encoding to model the sequence information. Since Transformer possesses an outstanding ability to capture long-range dependencies, it has achieved very good results in natural language processing problems. Therefore, in this paper, Transformer is used for the modeling and feature extraction of human regional data.

#### 2.2.1. Data Pre-Processing

The obtained timeseries data usually contain noisy elements, so the original series data need to be denoised. The more commonly used method is the sliding average method [26]. In this paper, the coordinate (*x*, *y*) data of every three adjacent frames of the same joint are used as a set. If the complete data of each joint has *m* frames, the averaging process divides the set into {*f*_1_, *f*_2_, *f*_3_}, {*f*_2_, *f*_3_, *f*_4_}, …, {*f_k_*_−1_, *f_k_*, *f_k_*_+1_}, …, {*f_m_*_−2_, *f_m_*_−1_, *f_m_*}. The set of each joint after denoising is {*F*_1_, *F*_2_, …, *F_m_*_−2_}, where *i* = 1, …, m−2:

(1) $$F_{i}=\frac{f_{k-1}+f_{k}+f_{k+1}}{3}$$

The set of outputs of all joints is {*J*_1_, *J*_2_, …, *J*_17_}.

#### 2.2.2. Regional Division

**Figure 2:**
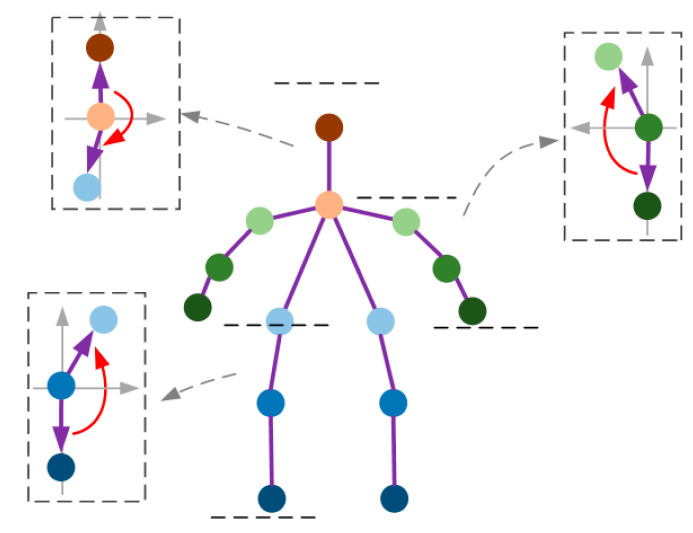
Human joint point labeling position.

In order to improve the processing efficiency, this paper divides the joint data into small regions by parallel segmentation of the regions represented by the human body according to the relationship between the left and right limbs. As shown in Figure 2, this paper selects 14 joints that best represent the human gait and posture characteristics as the research objects.

As shown in Table 1, every three adjacent joints were divided into one region. 14 topologically connected joints were divided into a total of 18 regions.

We focus on three human characteristics in one area, namely joint vector, limb length and joint angle. During walking, the joint vector *v*, limb length *l*, and joint angle θ calculated by Equations (2)–(4) will also change as the position of each joint changes. Although the vectors, angles, and lengths are calculated from the coordinate data of the joint points, we still hope that we can find the patterns of gait motion from the sequence data of different aspects. Where (*x*, *y*) are the pixel coordinates of the joint points,

(2) $$\vec{v}=\left(x_{k+1}-x_{k},y_{k+1}-y_{k}\right)$$

(3) $$l=\sqrt{{\left(x_{k+1}-x_{k}\right)}^{2}+{\left(y_{k+1}-y_{k}\right)}^{2}}$$

(4) $$\left\{ \begin{array}{ll} \theta =\arctan\frac{y_{k+1}-y_{k}}{x_{k+1}-x_{k}} & ,\;x_{k+1}\neq x_{k}\\ \theta =\frac{\pi }{2} & ,\;x_{k+1}=x_{k} \end{array}\right.$$

**Figure 3:**
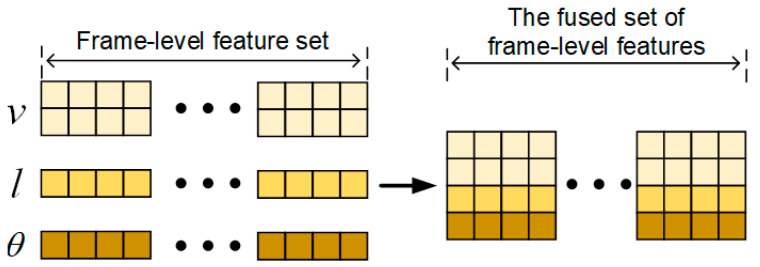
Frame-level feature stitching.

The above three kinds of data can be extracted from each image frame. In order to feed the time-series feature extraction network uniformly, we combine the three kinds of data by vector concatenation as shown in Figure 3.

#### 2.2.3. Regional Time-Series Coding Model

**Figure 4:**
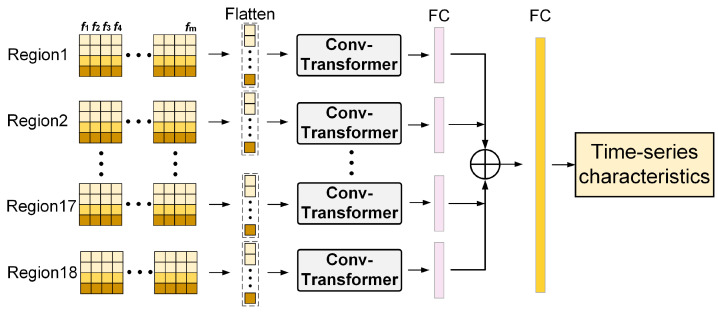
Transformer-based regional time-series coding model.

In this paper, a regional timeseries coding model is designed based on Transformer for extracting regional timeseries features. The structure of the Transformer-based regional time-series coding model is shown in Figure 4.

**Figure 5:**
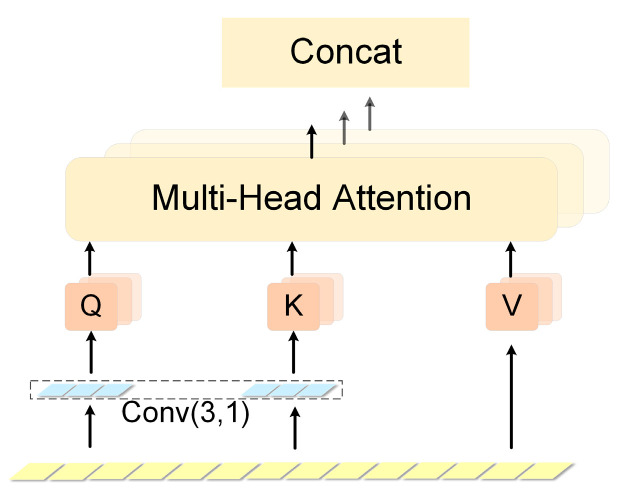
Convolutional self-attention.

Since both the encoder and decoder are networks based on the self-attention mechanism, the Transformer has a large spatial complexity in the computation. Meanwhile, the original Transformer is not sensitive enough to local information, making the model not very good at handling outliers. In order to solve the above problems, we used a method from the literature [27] and modified it in the self-attention module. This was done by first processing the input data using a convolution of size greater than 1 in the computation of Query and Key, so that attention could focus more fully on local contextual information. The convolution self-attention layer is shown in Figure 5.

First, the computed and concatenated data are fed into separate Conv-Transformer models according to the divided regions. For each region, the stitched data is 4 × *m* and expanded as a 4 × *m* × 1 one-dimensional vector. In Conv-Transformer, the data processed by a convolution kernel of size (3, 1) and step size 1 are used as a Query-Key for the matching calculation:

(5) $$\mathrm{Attention}(Q,K,V)=\mathrm{softmax}\left(\frac{Q\cdot K^{Τ}}{\sqrt{d_{k}}}\right)V$$

where *Q* is Query, *K* is Key, and *V* is Value, the arithmetic square root of the length of the sequence data vector.

The time-series feature vectors output by the self-attention module are concatenated into one region of time-series data features. The time-series features of multiple regions are finally concatenated and expanded again.

### 2.3. GAN-Based Network for Cross-View Gait Image Data Generation

**Figure 6:**
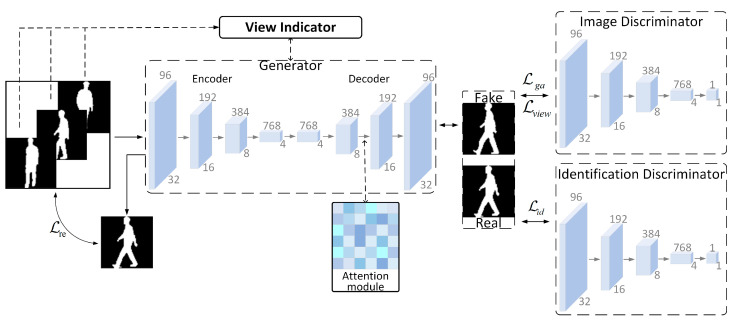
The overall structure of GAN-based multi-view gait image generation network.

In real life, acquiring multiple views and continuous and complete videos of human gait is very difficult. Existing gait datasets are often acquired in a laboratory setting. The subject is in a simple, empty environment with a simple background, and the subject’s walking state is captured by setting up cameras with multiple views. We wanted to be able to recognize human gait in the presence of partially missing or shorter-duration video input. To expand the gait data with algorithms, based on ideas from the literature [28], we use generative adversarial networks for gait data generation to expand the gait data set. However, while gait motion is continuous, gait images are acquired in a discrete manner. Therefore, it is difficult to achieve completely correct matching of image sequences of the same gait motion process under different views. In order to avoid large deviations, an unsupervised generative adversarial learning method is used in this paper. Meanwhile, for the generation of multiple views, only a single generator and discriminator are trained to complete the mapping of multiple views in order to avoid the overfitting problem caused by using a large number of convolutional neural networks. The overall structure is shown in Figure 6.

The fake images are generated through a generator and discriminator confrontation consisting of a convolutional neural network. The generator is used to process the input image data x and view information *v*, learn the distribution of the original data at a specific view, and generate the fake image y. To improve the quality of image generation, this paper adds a self-attention computation module to the generator network. The discriminator is used to estimate the probability that the corresponding input is real or fake. In the adversarial process, the goal of the generator is to map, as much as possible, the same distribution of real image data to send to the image discriminator for estimation. It is very important to keep the identity information during the cross view gait image generation process. Therefore, we propose an identity discriminator based on GaitGAN to distinguish the generated image identity information by training on identity loss. In order to make the images reconstructed by the generative adversarial network match the real images as closely as possible, a smooth L1 loss function [29] is introduced in this paper for maintaining the usability of the generated images. During the overall training of the network, the minimization and maximization of the adversarial loss functions are relied upon to constrain the generators and discriminators to

(6) $$\underset{G}{\min}\underset{D}{\max}ℒg_{a}=E_{y∼p_{data}(y)}[\log D(y)]+E_{x\sim P_{data}(x),v\sim P(v)}[\log(1-D(G(x,v)))]$$

In Equation (6), *G*(*x*,*v*) is a function of the generative network and *D*(*y*) is a function of the discriminant network; $ℒg_{a}$ is a value function characterizing the degree of difference between the real image data and the generated image data; the role of max is to hold the generative network *G* so that the discriminative network *D* maximizes the discrimination of the given data as true or false; and the role of min is to hold the discriminative network *D* so that the generative network minimizes the difference between the true samples and the generated samples.

The training process is divided into two stages. Firstly, the discriminator used to determine whether it is a true sample or a false sample is trained. When training the discriminator, the function of *D* is separated from Equation (6) and optimized using the gradient descent method. The loss function is

(7) $${ℒ}_{D}=-E_{y∼p_{data}(y)}[\log D(y)]-E_{x\sim P_{data}(x),v\sim P(v)}[\log(1-D(G(x,v)))]$$

Secondly, when training the generator, the function of *G* is separated from Equation (6) and optimized using the same gradient descent method. The loss function is

(8) $${ℒ}_{G}=E_{x\sim P_{data}(x),v\sim P(v)}[\log(1-D(G(x,v))]$$

#### 2.3.1. Generator Networks and Cyclic Reconstruction Loss

The generator network is based on the generator structure in GaitGAN, with the introduction of a self-attention module. First, the generator accepts a vector of gait images as input, which is processed by an encoder consisting of multiple convolutional kernels of size 4 × 4 and a pooling layer of step size 1. This is followed by a decoder consisting of multiple deconvolution layers and an attention module to generate the gait data. The final generated fake data are used to deceive the discriminator model and will be gradually improved during the training process, guided by the view indicator to generate more realistic data. Among them, attention is computed in the same way as introduced in the previous section.

During the generation of image data, it is necessary to retain other information in addition to views and identities, which is information that needs to be retained. For example, the walking status (wearing a coat, carrying a bag, etc.) of the same subject in the same view may be different. In order to keep the style of the generated image consistent with the original image as much as possible and to make the reconstructed generated image more stable, a pixel-level Smooth L1 loss is used in this paper. The pixel error between the generated image *x* and the real image y is minimized by training the Smooth L1 loss as follows:

(9) $${ℒ}_{\mathrm{re}}\left(G(x),\;y\right)=E_{x∽P_{data}(x),v∽P(v)}\left[\frac{1}{H\times W}\sum_{i=1}^{H\times W}\{\begin{array}{c} 0.5*{\left|y_{i}-G(x_{i})\right|}^{2},\;\;if\left|y_{i}-G(x_{i})\right|<1\\ \left|y_{i}-G(x_{i})\right|-0.5\;,\;\;otherwise \end{array}\right]$$

#### 2.3.2. View Classification Loss and Identification Loss

For a given gait silhouette input *x*, the generative adversarial network can generate a gait silhouette image of a specific view guided by a view indicator *v*. When the discriminator receives the image data, the discriminator will determine whether the input data is from the real sample or the data generated by the generator and classify the views of that image data. To optimize the discriminator, this is achieved by minimizing the objective function

(10) $${ℒ}_{view}=E_{x\sim P_{data}(x),v\sim P(v)}[\log D_{view}(G(x,v))]$$

Meanwhile, in order to avoid the traditional problem of generative adversarial networks ignoring the continuity between frames in the image reconstruction process, which leads to the identity loss problem in the generated multi-view gait images, we use an identity discriminator to increase the model stability. The image data in the real sample and the corresponding generated image data are fed into the identity discriminator as a set of training samples. The identity discriminator will calculate the probability that this set of data is the gait image data of the same person. This is achieved by optimizing the objective function

(11) $${ℒ}_{id}=E_{y∼p_{data}(y)}[\log D_{id}(y)]-E_{x\sim P_{data}(x),v\sim P(v)}[\log(1-D_{id}(G(x,v)))]$$

Combining each of the above optimization objectives, the total loss function for generating multi-view gait silhouette using GAN is:

(12) $${ℒ}_{\mathrm{all}}\left(G,D\right)=\lambda_{1}{ℒ}_{ga}+\lambda_{2}{ℒ}_{view}+\lambda_{3}{ℒ}_{id}+\lambda_{4}{ℒ}_{re}$$

where $\lambda_{i}$ is a hyperparameter that can be adjusted during the optimization process to control the weights of different loss functions in the overall network impact.

### 2.4. Gait Silhouette Feature Extraction

**Figure 7:**
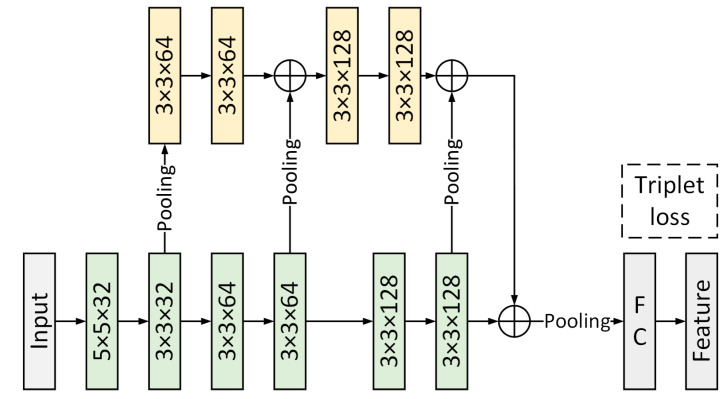
Feature extraction network for silhouette branching.

In this paper, the gait silhouette feature extraction is based on the GaitSet convolutional neural network structure, as shown in Figure 7. The input of the network is the expanded gait silhouette dataset. The feature extraction backbone consists of 6 convolutional layers. The branches of the network are used to fuse the time-series features of the silhouette images. The two features obtained from the backbone and branches are concatenated and mapped through fully connected layers to obtain the gait contour features. In order to fully use this network for feature extraction, the input image is cut, scaled, and cropped to obtain a gait contour map of size 64 × 64.

### 2.5. Feature Fusion Module Based on Bilinear Matrix Decomposition Pooling

After the input data are passed through the time-series branch network and the silhouette branch network, the body regional time-series data features $f_{t}$ and silhouette features $f_{o}$ of the human walking process are obtained, respectively: $f_{t}\in {\mathbb{R}}^{m\times 4\times 18}$ and $f_{o}\in {\mathbb{R}}^{15872\times 1}$. The common methods of feature-level fusion are weighted average, tensor concatenation, etc. In the two-branch network of the algorithm in this paper, the dimensionality of the output features is very different due to the different structures of the time-series branch network and the silhouette branch network. Therefore, the traditional feature fusion methods are not applicable to the network of the algorithm in this paper. In order to make full use of the different types of features extracted from the dual branch network, this paper adopts a feature fusion method based on bilinear matrix decomposition pooling.

Bilinear pooling has gained more attention from researchers since it was proposed by Lin et al. [30] for fine grained classification. For the feature fusion process from two feature extractors, it is called Multimodal Bilinear Pooling (MBP). The process of bilinear pooling is to obtain a feature matrix by bilinearly fusing (multiplying) two features at the same position, and then sum pooling the feature matrices at all positions, and finally expanding the pooled matrix into a vector. After performing matrix normalization and L2 normalization operations on this vector, the fused features are obtained. However, the original bilinear pooling suffers from the problem that the dimensionality of the fused features is too high. Some researchers have improved on the MBP [31,32,33]. Based on a priori knowledge, we designed the feature fusion module based on the introduction of bilinear matrix decomposition and horizontal pyramidal pooling.

For the time-series data features $f_{t}$ and silhouette features $f_{o}$, the bilinear pooling can be defined as

(13) $$Z_{i}=f_{t}{}^{T}W_{i}f_{o}$$

where $W_{i}\in {\mathbb{R}}^{m\times 4\times 18\times 15872}$ is a projection matrix and $Z_{i}$ is the output of the bilinear pooling model. The projection matrix is decomposed into two low-rank matrices:

(14) $$Z_{i}=f_{t}{}^{T}U_{i}V_{i}f_{o}$$

Expanding the decomposed matrix dimensions into the sum form,

(15) $$Z_{i}=\sum_{d=1}^{k}f_{t}{}^{T}u_{d}v_{d}{}^{Τ}f_{o}=1^{Τ}\left(U_{i}{}^{Τ}f_{t}{}^{T}⊙V_{i}{}^{Τ}f_{o}\right)$$

where *k* is the dimensionality of $U_{i}=\left[u_{1},\ldots ,u_{k}\right]$ and $V_{i}=\left[v_{1},\ldots ,v_{k}\right]$, $1^{Τ}$ is the k-dimensional all 1 vector, and ⊙ denotes the Hadmard product.

The decomposed feature matrix is sent to the horizontal pyramid [34] for dimensionality reduction. The horizontal pyramid used in this paper is divided into four scales of 1, 2, 4, 8. The input feature levels are partitioned into hierarchical regions of feature data according to the pooling of different scales. The segmented data is denoted by $Z_{n}^{m}$, which can be understood as the feature data of the *m*th region in the *n*th scale. The feature vector of the pyramid output is represented by $T_{n}^{m}$:

(16) $$T_{n}^{m}=\mathrm{avgpool}\left(F_{n}^{m}\right)+\mathrm{maxpool}\left(F_{n}^{m}\right)$$

Afterwards, the $T_{n}^{m}$ downscaling is performed again by a 1 × 1 convolution. The mapping is performed using a fully connected layer, and the resulting feature vectors are used for classification.

## 3. Experiment

To evaluate the effectiveness of the time-series feature extraction network, the silhouette feature extraction network, and the two-branch feature fusion network in gait recognition, we conducted experiments on the OUMVLP-Pose dataset [35] and the CASIA-B dataset [36], as well as on data collected in real scenes.

### 3.1. Datasets

In order to verify the feature extraction capability of the previously mentioned regional time-series coding network and the effect of the gait silhouette image generation network, we selected the OUMVLP-Pose gait recognition dataset with human keypoint location sequence labels and the large gait dataset CASIA-B, consisting of gait silhouette maps for the single-branch network, respectively. Due to the lack of a public dataset containing both human keypoint annotations and gait silhouettes, for the validation of the fusion effect of the two-branch network, we acquired real-world videos of people walking. Based on the recorded videos, we created a small dataset of gait recognition containing both human keypoint data and gait silhouette images.

#### 3.1.1. Public Datasets

**Figure 8:**
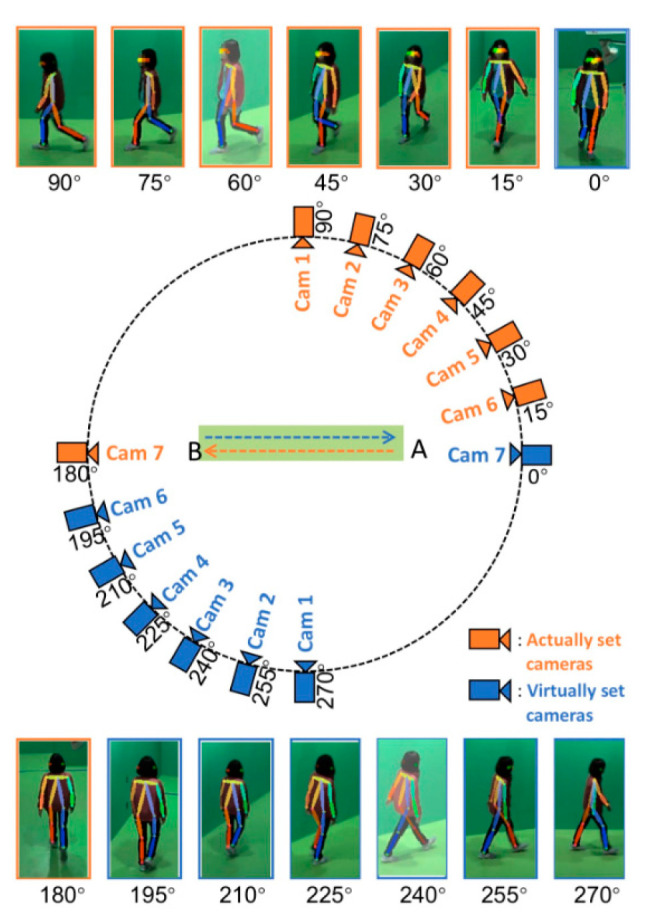
Collection settings for the OUMVLP-Pose dataset.

The OU-MVLP dataset is a large multi-view pedestrian dataset created by Osaka University, Japan. The dataset contains 10,307 walkers, including 5114 males and 5193 females, distributed in different age groups. The dataset contains a total of 14 views with 15° intervals between the views, and OUMVLP-Pose is built on top of OUMVLP. The builder of the dataset used pre-trained models from OpenPose [37] and AlphaPose [38] to extract the human skeletal point location information from the RGB images of OUMVLP. Figure 8 shows the schematic diagram of the OUMVLP-Pose dataset acquisition provided by the OU-ISIR biometric database website.

**Figure 9:**
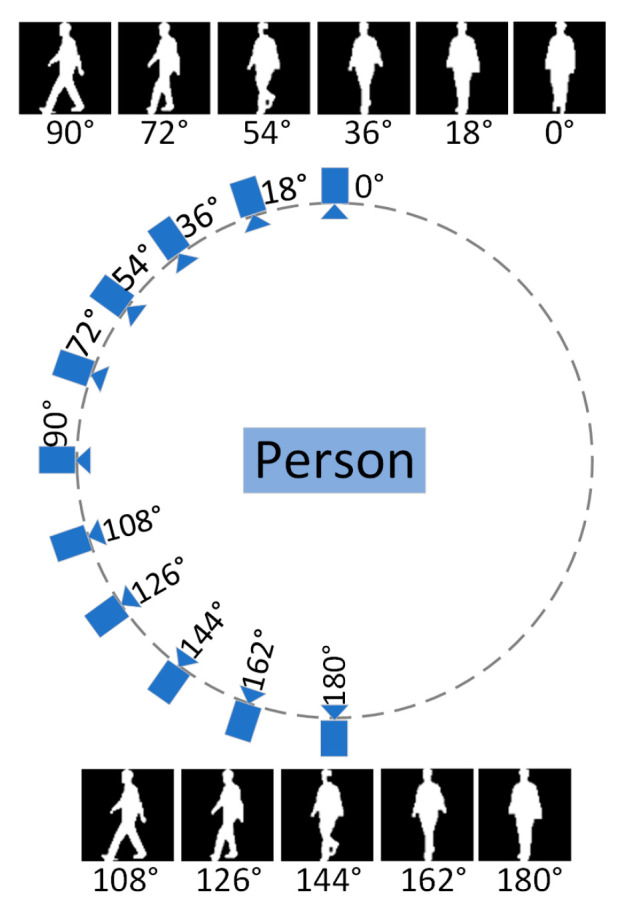
Gait silhouette images in the CASIA-B dataset.

The CASIA-B dataset contains a total of 124 walkers and three walking states, including normal walking (NM) with six sequences per person, walking with a bag (BG) with two sequences per person, and walking while wearing a coat (CL), with two sequences per person. Each sequence for each pedestrian has 11 observed viewing angles with an angle range (0°, 18°, 36°, …, 180°) at 18° intervals. Figure 9 shows a schematic of the gait silhouette acquisition environment in the CASIA-B dataset.

#### 3.1.2. Test Data in Real Scenarios

**Figure 10:**
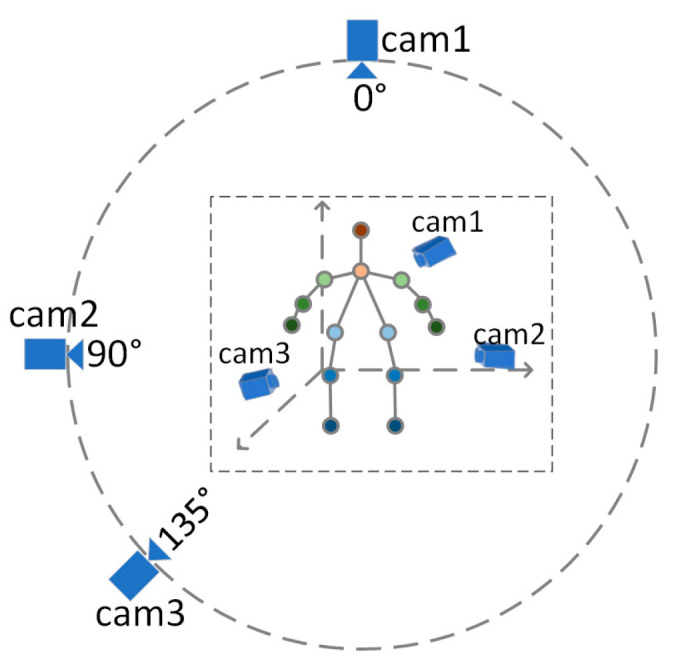
View setup for gait data acquisition in the laboratory environment.

In order to evaluate the two-branch fusion model presented in the previous chapter, gait data were collected in a realistic scenario. The acquisition was performed by setting up a multi-view camera in a laboratory environment with nine subjects walking at a uniform speed on a walking machine. In order to simulate the process of real-life surveillance cameras on people, the camera views were located at 0°, 90°, and 135° of the subject’s body (0° directly in front of the body and increasing counterclockwise). Figure 10 shows the schematic diagram of the acquisition environment for the test data.

### 3.2. Experimental Environment and Setup

The experimental environment is a Windows 10 operating system and Python 3.7 IDE; the deep learning framework uses Pytorch; to improve the model computing efficiency, an NVIDIA RTX3080Ti is used and CUDA11.0 and the corresponding cuDNN deep learning acceleration library is installed.

In the regional time-series coding branch network training, 20 walkers were randomly selected from the training sample in each iteration, and then 10 sequences were randomly selected from the data of each walker. After that, 20 consecutive frames were randomly selected from each sequence as the input data. The network used the Adam optimizer and the initial learning rate was set to 0.0002. In the data expansion network for silhouette images, firstly, the effectiveness of the generative adversarial network for generating images was evaluated. This was followed by a gait recognition test using the expanded dataset. During the training process, a total of 80,000 iterations were performed. The initial learning rate was 0.0001, and the learning rate was decayed to 0.1 times at the 60,000th iteration. The threshold distance of triplet loss was set to 0.2. The data set was divided by the large-sample training (LT) method [16]. Data from the first 74 walkers were used for training, and data from the last 50 were used for testing.

### 3.3. Experimental Results and Analysis

This section presents the results of the experimental analysis of single-branch and two-branch fusion networks.

#### 3.3.1. Recognition Effect Based on the Regional Time-Series Coding Network

**Figure 11:**
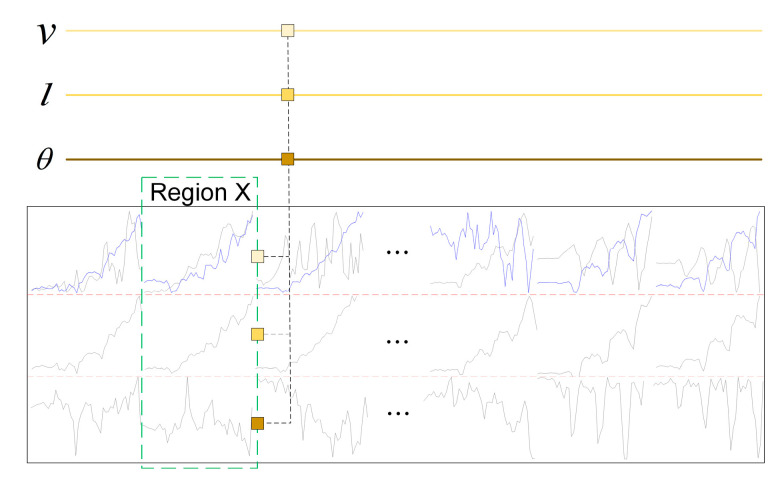
The change curves of joint data.

**Figure 12:**
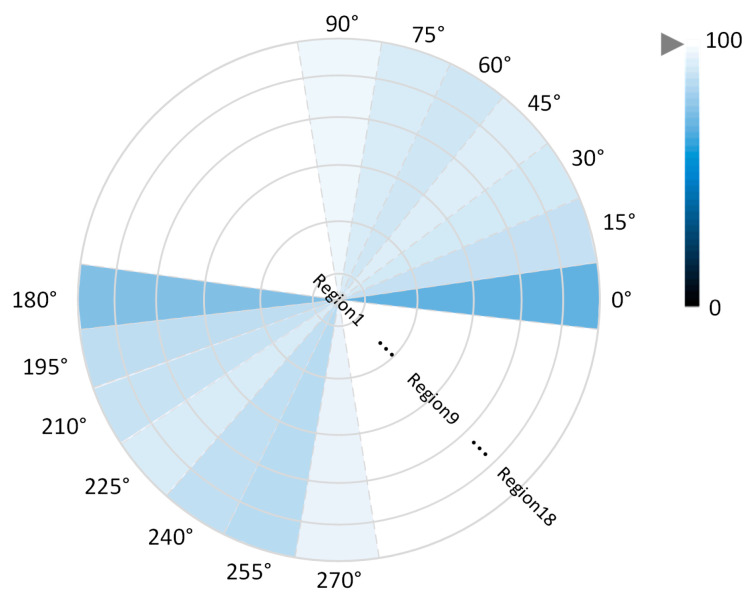
Comparison of the results of wearable and non-wearable measurement methods.

**Figure 13:**
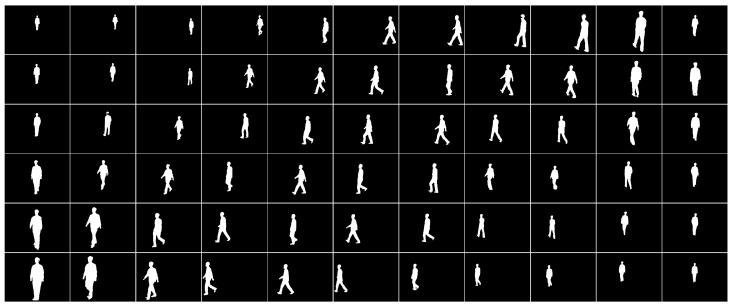
Part of the generated image dataset.

There are 18 human-joint-annotated positions in the OUMVLP-Pose dataset, but the left eye, right eye, left ear, and right ear data are not significantly helpful for gait recognition. Therefore, based on the human joint position settings in this paper, we used the annotation data of joints 0–13 extracted by the OpenPose algorithm in the OUMVLP-Pose dataset. Figure 11, Figure 12 and Figure 13 show the change graphs of 50 frames of data in randomly selected individual regions calculated from 18 human regions divided according to Table 1, respectively. Figure 11 shows the variation curves of vectors, inter-joint distances, and joint angles obtained for each region.

Table 2 shows the accuracy of the time-series feature extraction branch designed in this paper on the OUMVLP-Pose dataset. We extracted the keypoint data annotated in the OUMVLP-Pose dataset into a uniform csv format data list in the form of sequence data as the input of the temporal feature extraction branch. Compared with LSTM and Transformer networks, which are commonly used for processing sequence data, the network we designed achieved relatively better results. In particular, the accuracy of Rank-1 is higher for 90° and 270°. The reason for this situation may be that when the OUMVLP-Pose dataset uses the OpenPose and AlphaPose algorithms to identify human keypoints, these two side views observe the human joints more obviously, which makes the extracted pixel location information of keypoints more accurate. To validate this idea, we used Noitom’s motion-capture suite to obtain real-time data streams of the movements from the accompanying software. The acquired data were normalized and calculated to obtain the human keypoint position information at the same pixel position coordinates as the OUMVLP-Pose dataset. Figure 12 shows the comparative analysis of the human keypoint data captured by the sensor during the motion and the human keypoint data obtained using OpenPose and AlphaPose algorithms in the OUMVLP-Pose dataset. The evaluation index used for the comparison is PCKh, which is the proportion of the normalized distance between the keypoint data detected using the physical method and the data labeled in the dataset that is less than a set threshold, using the head distance as the normalized reference. The data from PCKh@0.5 is considered correct when the distance between the positions of the two keypoints is less than 50% of the diagonal length of the bounding box of the head.

In Figure 12, the PCKh results are mapped into the HSV color space, and the change in the value of PCKh is indicated by the color shade. The darkest color indicates that PCKh is equal to 0, and the lightest color indicates that PCKh is equal to 100. In this paper, we compared the data measured by the wearer, the data recognized by OpenPose in the dataset, and the data recognized by AlphaPose in the dataset, and the results shown in Figure 12 were obtained after calculating and averaging the two. The difference between the data in the dataset and the real data can be seen in Figure 12. It also confirms the problem related to the recognition effect proposed above.

#### 3.3.2. Effect of the Multi-View Gait Image Generation Network

This section discusses the effect of the generation of our proposed gait silhouette images. Figure 13 shows the generated fake images trained from the real images in the CASIA-B dataset.

In order to show the effectiveness of the image generation method proposed in this paper, the distribution of the generated data was evaluated using the Inception Score [39]. Inception Score (IS) is a KL divergence (relative entropy) calculation of the data:

(17) $$\mathrm{IS}\left(G\right)=\exp\left(E_{x\sim P_{g}(x)}\mathrm{KL}\left(p\left(y|x\right)\|p\left(y\right)\right)\right)$$

where p(y|x) is the probability of the category output for a given generated image x, after feeding it into a pre-trained Inception classification network [40], and p(y) is the edge distribution, which represents the expectation of the probability of the category output by this pre-trained classification network for all generated images. If the generated image contains meaningful and clearly identifiable targets, the classification network should determine that image as a specific category with a high confidence level, so p(y|x) should have a small entropy. In addition, for the generated images to be diverse, p(y) should have a large entropy. If p(y) has a large entropy and p(y|x) has a small entropy, i.e., the generated images contain very many categories, and each image has a clear and high confidence category, then p(y|x) and p(y) have a large KL scatter.

Based on the above analysis, the IS entropy value is used to determine the degree of dispersion of the generated data relative to the standard data, using the data distribution of the gait silhouettes in each view in the data set as a benchmark. In the IS calculation, the larger the IS value, the closer the generated data is to the ideal state. Also, the Kernal MMD [41] and Wasserstein distance [42] methods were used in this paper to evaluate the quality of the generated images, and the evaluation results are displayed in Table 3.

**Figure 14:**
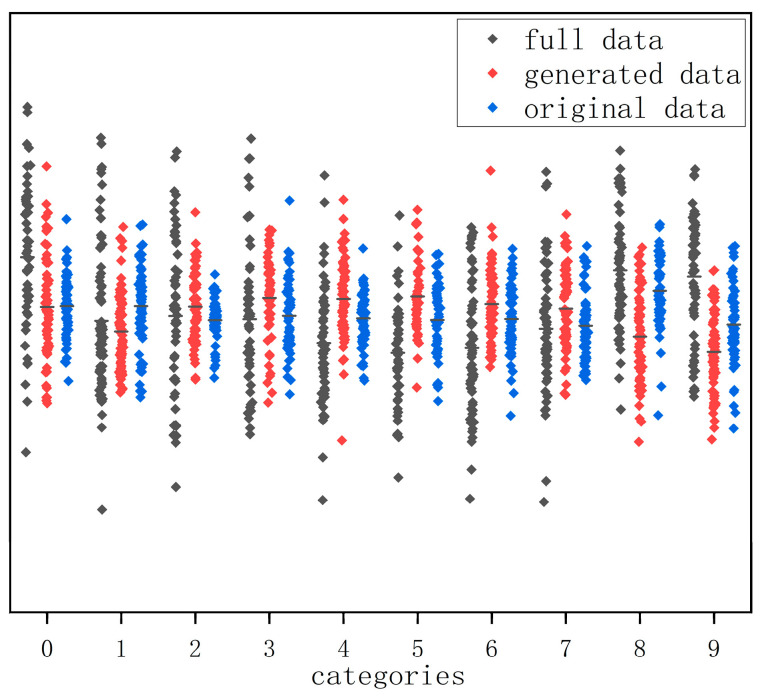
Distribution of some original and generated data.

Figure 14 shows the gait data of 10 people randomly selected from the original dataset, the generated dataset, and the fused dataset, respectively. From Figure 14, it can be seen that the distribution of gait silhouette data generated using the algorithm of this paper has a similar pattern to that of the same kind in the dataset and achieves the purpose of expanding the gait dataset in terms of quantity.

Finally, we conducted tests using the original dataset as well as the expanded gait silhouette dataset, and the results are shown in Table 3. From Table 4, it can be seen that the accuracy of recognition is higher after expanding the dataset due to the increase in data volume. However, compared to the 90° side view, the accuracy improvement is more obvious for the other views. This indicates that the side view exhibits richer and clearer silhouette information. Therefore, the silhouette data from each view can also be relearned afterwards and all converted to the 90° view for testing using gait energy images (GEI).

To better demonstrate the effectiveness of our design, we used the same method as GaitGAN to train and test our designed gait silhouette image generation network. The dataset used was CASIA-B and was divided into training set, gallery set, and probe set. In the experimental design of GaitGAN, three states, NM, BG and CL, were included. The gait data of the first 62 subjects were put into the training set, and the gait data of the remaining 62 subjects were put into the test set. In the test set, the first four sequences of each subject in one state were put into the gallery set, and the last two sequences were put into the probe set. By putting the data from the gallery set into the model, the corresponding features were output, and then the data from the probe set were also put into the model to get the corresponding features. The two features were compared and the corresponding similarity results were output. Table 5, Table 6 and Table 7 show the results obtained after image generation using our gait silhouette image generation adversarial network and performing feature extraction and matching compared with the results obtained using GaitGAN. As can be seen from the table, our method achieves higher recognition rates than GaitGAN in most views. However, in the BG and CL states, the recognition rate of some views of GaitGAN is higher than that of our method. After the experiments, it can be seen that our gait silhouette graph generation network needs to be optimized compared to the GEI generation method of GaitGAN when strong disturbances are included. In future work, we will consider the simultaneous generation of silhouette images under different viewpoints as well as the synthesis of GEI under specific views to provide more sufficient data for improving the gait recognition process.

#### 3.3.3. Testing of the Fusion Model in Real Scenarios

The testing in real scenarios was divided into two parts: quantitative assessment and qualitative assessment.

**Figure 15:**
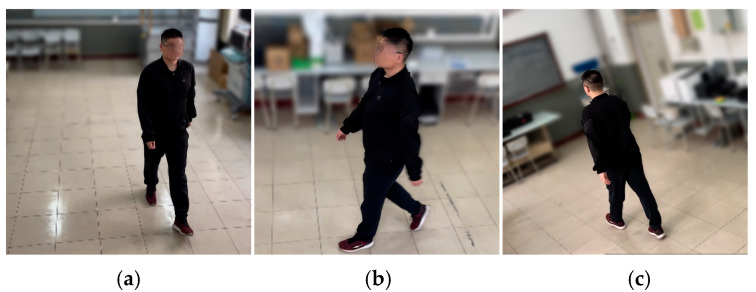
Scene of data collection. (**a**) View of 0° (**b**) View of 90°. (**c**) View of 135°.

**Figure 16:**
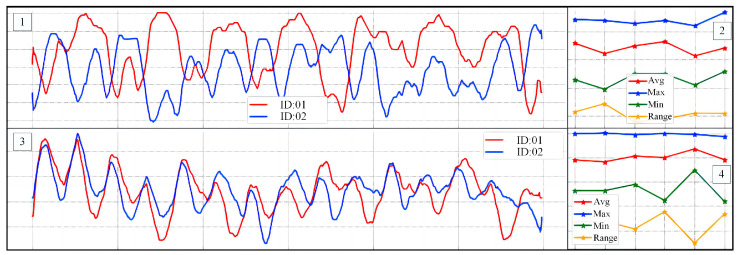
Comparison of trends in movement characteristics of different volunteers.

First was the quantitative evaluation part. By setting up cameras under three views of 0°, 90°, and 135° in an indoor environment, the data of keypoints of the human body were collected using OpenPose and the data under the three views was averaged. The silhouette extraction was performed by background subtraction. Figure 15 shows our gait data collection in different views. In accordance with our laboratory regulations and data privacy instructions, we defocused the background of the images and mosaicked the volunteers’ faces. In addition, we visualized the extracted feature data for characterizing the relevant motion patterns. This is shown in Figure 16. 

In Figure 16, region 1 shows the curve obtained after min-max normalization of the fused feature vectors, and the data in region 2 are the first five larger and the last five smaller data extracted from region 1. Region 3 is the curve obtained after Z-score normalization of the fused feature vectors, and the data in region 4 are the first five larger and the last five smaller data extracted from region 3. From Figure 16, we can see that different walkers show different movement patterns in their bodies while walking. According to these patterns, we can effectively identify the walkers in the video.

To verify that the module we designed has better feature fusion effects, we chose three fusion methods—Concatenation, Squeeze-and-Excitation Networks (SENet) [43], and Feature Pyramid Network (FPN) [44]—for comparison. The experimental results are shown in Table 8. Compared with Concatenation, the method based on bilinear pooling decomposition can utilize the acquired feature information more effectively and reduce the data loss due to information fusion. Compared with SENet, the computational process of our method is simpler. It can still obtain good feature fusion results with reduced computing resources. FPN is a commonly used feature fusion method that maintains high quality information during feature fusion by adding lateral connections to the feature pyramid at different levels. Our method is a bit more complex than FPN, mainly due to the addion of a bilinear matrix decomposition computational process before feeding into FPN. The purpose of this is to utilize the feature information as much as possible and to reduce the dimensionality of the computed fused features by horizontal pyramid pooling. Therefore, our method achieves better results than using only FPN.

The Rank-1 and Rank-5 accuracy of recognition with data collected in real scenes is shown in Table 9. Rank-1 accuracy is the percentage of the number of predicted category labels with the maximum probability that the true label is equal to the total number of samples. Rank-5 accuracy is the percentage of the predicted category with the maximum probability that one of the five categories is the same as the true label, and the prediction result is true.

**Figure 17:**
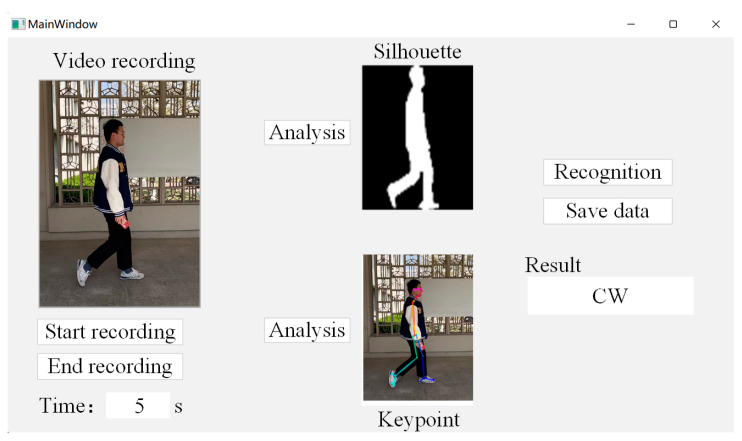
Interactive interface of gait recognition system.

Finally, there is the qualitative evaluation part. We used a short-time video taken for recognition effect testing, and the duration of the pedestrian walking video is 5 s. In order to systematize the recognition process, we designed the upper computer interface of the gait recognition system using PyQt5, as shown in Figure 17.

By recording the video and analyzing it, the feature data extracted from the two branch networks are fused to achieve human-gait-based identity recognition. The time duration and effect of each stage of the recognition process are shown in Table 10, which proves the feasibility and practicality of the design of this paper.

## 4. Discussion

Gait recognition technology has a wide range of prospects in the real world. In practical applications, the large amount of data required for gait recognition has been an important factor affecting the recognition results. In this paper, we have considered a combination of human time-series feature extraction and gait data expansion to achieve gait recognition with less data. In Section 3.3.1, we analyzed the regional time-series data in order to obtain the motion pattern of human walking and to observe the effect of our designed time-series feature extraction network. By visualizing and analyzing the joint vectors, inter-joint distances, and joint angles, we found that the distribution of our regional time-series data has different patterns when a person is walking. This laid the foundation for our next step of quantitative analysis. After comparing with the commonly used time-series feature extraction networks, we found that the time-series feature extraction network we used has better results. To evaluate the effectiveness of the multi-view gait silhouette generation, we performed KL scatter analysis by calculating the Inception Score and proved that our gait silhouette generation network is effective. After classification using a unified feature extraction algorithm, it was also demonstrated that the gait dataset after data expansion showed more significant identity feature information than the original dataset. Tests in real scenarios also provided proof of the effectiveness of our approach. In this paper, our main work is the extraction of time-series features of human motion and the data expansion of gait silhouette images. In future work, we will conduct a more detailed study, including the optimization of the time-series feature extraction network and the gait silhouette feature extraction network, especially the design of the Transformer-based feature extraction network. For example, the CSTL [45] network constructed based on Transformer and the Significant Spatial Feature Learning (SSFL) module has achieved good results in feature extraction of gait silhouette image using the global relationship modeling capability of the proposed network. Also, we can add a regularization method similar to ReverseMask [46] to improve the feature extraction capability for gait images, and to improve the accuracy of gait recognition.

## 5. Conclusions

For the problem of low accuracy of gait recognition caused by incomplete and insufficient gait data under short video input, this paper designs a method to fuse time-series branch and contour branch data. By analyzing the relationships between human limbs during motion, the regions used to characterize human motion patterns are defined. The time-series features are extracted from the data changes of the keypoints of the human body in each region. The method of CNN combined with Transformer is used for temporal feature extraction. This method solves the problem that Transformer has the ability of long-range modeling but is insensitive to local information. In this paper, the OUMVLP-Pose dataset is used to test the temporal branching network. The test results show that the feature extraction capability of the time-series feature extraction branch designed in this paper is stronger than that of the general time-series data processing network. In order to expand the gait silhouette data for recognition, this paper designs a generative adversarial network that generates gait image data according to the distribution pattern of the input data. The silhouette branch was tested using the silhouette maps of the CASIA-B dataset. The effectiveness of the generative adversarial network designed in this paper is proved according to the IS entropy value and the distribution law of the generated data. In order to fully combine the time-series data features and contour data features, a feature fusion module based on bilinear matrix decomposition pooling is designed in this paper. This feature fusion module fuses the feature data of two different dimensions on the basis of fully preserving the original features. In this paper, the bifurcated fusion model is tested under real scenarios in terms of both qualitative and quantitative evaluation, and the results show that the model we designed has high accuracy. The designed upper computer interface can integrate the recognition process, which makes the design of this paper have more practical feasibility.

## Figures and Tables

**Table 1:** Regional division method of human body representation.

Region	Joints	Region	Joints
1	Head + Spine + Left Shoulder	10	Left Shoulder + Spine + Right Shoulder
2	Head + Spine + Right Shoulder	11	Left Shoulder + Spine + Left Hip
3	Head + Spine + Left Hip	12	Left Shoulder + Spine + Right Hip
4	Head + Spine + Right Hip	13	Right Shoulder + Right Elbow + Right Wrist
5	Spine + Left Shoulder + Left Elbow	14	Right Shoulder + Spine + Left Hip
6	Spine + Right Shoulder + Right Elbow	15	Right Shoulder + Spine + Right Hip
7	Spine + Left Hip + Left Knee	16	Left Hip + Spine + Right Hip
8	Spine + Right Hip + Right Knee	17	Left Hip + Left Knee + Left Ankle
9	Left Shoulder + Left Elbow + Left Wrist	18	Right Hip + Right Knee + Right Ankle

**Table 2:** Classification accuracy of time-series data extracted from the OUMVLP-Pose dataset.

	Accuracy					
	Openpose			Alphapose		
	LSTM	Transformer	Ours	LSTM	Transformer	Ours
0°	74.5	83.8	86.8	75.6	80.5	86.7
15°	81.8	82.2	88.9	77.3	81.3	87.5
30°	77.1	78.8	85.4	80.2	80.6	86.2
45°	81.5	80.7	80.9	82.0	82.7	81.5
60°	78.9	77.3	80.6	76.5	79.2	81.5
75°	81.1	83.0	81.7	80.2	85.0	81.0
90°	81.8	84.1	86.0	80.0	86.5	85.7
180°	79.0	82.5	87.8	76.2	81.4	85.6
195°	77.8	88.2	90.9	79.6	85.6	89.8
210°	73.5	84.4	86.8	75.7	83.3	88.2
225°	80.7	82.0	85.8	81.6	81.2	86.5
240°	72.1	79.2	85.4	78.3	80.5	84.6
255°	79.3	83.5	86.3	80.2	82.4	85.4
270°	79.3	85.3	90.6	81.6	86.5	89.5
Average	78.5	82.5	85.9	78.9	82.6	85.7

**Table 3:** Evaluation results of the generated images using different evaluation methods.

Inception Score	Kernal MMD	Wasserstein Distance
3.62 ± 0.07	3.75 ± 0.04	4.02 ± 0.04

**Table 4:** Rank-1 accuracy obtained by testing on various subsets of the CASIA-B dataset.

Rank-1 Accuracy						
Algorithm	GaitSet					
Dataset	Original Data			Full Data		
	NM	BG	CL	NM	BG	CL
0°	87.7	82.5	62.1	91.8	85.8	66.4
18°	90.2	85	73.4	95.9	90.2	75.4
36°	91.6	88.1	79.5	93.4	91.8	81.7
54°	90.6	85.5	78.3	94.9	88.7	77.3
72°	89.2	85.4	73.4	93.6	83.3	72.1
90°	90.2	82.5	72.3	91.7	81	73.1
108°	91.3	83.3	70.6	95	84.1	72.5
126°	92.6	87.2	72.4	94.8	92	73.5
144°	92.7	90.1	76.9	95.9	92.2	78.6
162°	93.6	90.5	66.4	92.8	94.4	68.4
180°	87.8	80.1	51.2	85.8	79	65
Average	90.7	85.5	70.6	93.2	87.5	73.1

**Table 5:** Recognition Rate of ProbeNM.

	GaitGAN	Probe Set View (Normal Walking)																					
Ours		0°		18°		36°		54°		72°		90°		108°		126°		144°		162°		180°	
**Gallery set view**	0°		100.0		79.03		45.97		33.87		28.23		25.81		26.61		25.81		31.45		54.84		72.58
		99.70		77.52		40.64		31.52		26.00		25.70		26.90		25.42		30.67		52.66		70.53	
	18°		78.23		99.19		91.94		63.71		46.77		38.71		37.90		44.35		43.55		65.32		58.06
		77.09		99.65		90.47		61.55		46.79		32.76		36.54		40.32		44.69		65.06		58.77	
	36°		56.45		88.71		97.58		95.97		75.00		57.26		59.68		72.58		70.16		60.48		35.48
		54.36		88.44		98.04		93.72		74.56		55.49		56.38		71.72		70.62		61.55		34.42	
	54°		33.87		53.23		85.48		95.97		87.10		75.00		75.00		77.42		63.71		37.10		22.58
		30.28		50.89		85.04		96.76		87.05		73.02		72.97		77.46		62.51		37.04		20.06	
	72°		27.42		41.13		69.35		83.06		100.0		96.77		89.52		73.39		62.10		37.10		17.74
		26.52		45.37		66.73		80.24		99.89		97.02		88.42		71.53		60.00		34.49		15.08	
	90°		22.58		37.10		54.84		74.19		98.39		98.39		96.77		75.81		57.26		35.48		21.77
		22.56		38.46		53.54		72.26		98.74		98.02		95.57		76.56		54.95		36.04		20.43	
	108°		20.16		32.26		58.06		76.61		90.32		95.97		97.58		95.97		74.19		38.71		22.58
		18.55		31.42		57.58		76.09		91.25		94.46		98.50		94.75		71.32		39.45		21.08	
	126°		29.84		37.90		66.94		75.00		81.45		79.03		91.13		99.19		97.58		59.68		37.10
		27.05		36.51		65.70		75.03		80.25		77.84		90.46		99.60		97.05		56.81		37.05	
	144°		28.23		45.97		60.48		66.94		61.29		59.68		75.00		95.16		99.19		79.84		45.97
		26.57		44.76		58.16		65.72		61.57		58.67		75.04		94.67		99.65		78.64		44.97	
	162°		29.03		34.68		36.29		25.00		19.35		16.13		20.16		37.90		51.51		76.61		41.94
		28.34		31.68		34.78		25.07		18.63		15.46		17.65		35.44		52.49		78.42		40.91	
	180°		42.74		28.23		24.19		12.90		11.29		11.29		14.52		21.77		30.65		49.19		77.42
		41.91		27.62		22.95		12.46		10.79		10.66		14.63		20.49		29.54		47.65		78.29	

**Table 6:** Recognition Rate of ProbeBG.

	GaitGAN	>Probe Set View (Walking with a Bag)																					
Ours		0°		18°		36°		54°		72°		90°		108°		126°		144°		162°		180°	
**Gallery set view**	0°		79.03		45.97		33.06		14.52		16.13		14.52		11.29		15.32		22.58		33.87		41.13
		78.02		44.59		30.46		15.42		15.16		11.65		11.32		13.79		21.86		31.35		40.65	
	18°		54.84		76.61		58.87		31.45		26.61		16.13		24.19		29.84		32.26		41.94		32.26
		52.59		78.63		56.75		30.59		25.81		16.34		24.94		27.96		30.76		40.19		32.46	
	36°		36.29		58.87		75.81		53.23		44.35		30.65		34.68		46.77		42.74		34.68		20.16
		35.68		56.72		75.06		52.68		43.95		27.96		35.49		45.81		40.36		33.91		20.04	
	54°		25.00		45.16		66.13		68.55		57.26		42.74		41.13		45.97		40.32		20.16		13.71
		23.86		44.69		65.72		67.79		55.60		41.75		40.69		44.68		40.35		17.67		13.49	
	72°		20.16		24.19		38.71		41.97		65.32		56.45		57.26		51.61		39.52		16.94		8.87
		19.57		23.74		36.94		40.57		66.25		55.87		58.61		50.46		40.57		17.56		8.70	
	90°		15.32		27.42		37.90		38.71		62.10		64.52		62.10		61.29		38.71		20.97		12.10
		9.70		18.94		36.25		37.46		58.30		62.81		60.70		59.60		37.76		15.44		10.33	
	108°		16.13		25.00		41.13		42.74		58.87		58.06		69.35		70.16		53.23		24.19		11.29
		15.79		25.06		40.74		41.52		58.07		58.05		70.59		69.76		53.62		23.69		10.44	
	126°		19.35		29.84		41.94		45.16		46.77		52.42		58.06		73.39		66.13		41.13		22.58
		18.52		27.56		41.44		45.21		45.76		50.00		57.17		73.46		65.17		41.66		22.75	
	144°		26.61		32.26		48.39		37.90		37.10		36.29		38.71		67.74		73.39		50.00		32.26
		25.60		31.47		46.25		37.69		35.94		36.42		37.85		66.79		70.15		45.70		31.58	
	162°		29.03		34.68		36.29		25.00		19.35		16.13		20.16		37.90		51.51		76.61		41.94
		28.64		33.54		35.48		26.45		20.49		16.08		19.46		38.05		50.77		75.15		40.85	
	180°		42.74		28.23		24.19		12.90		11.29		11.29		14.52		21.77		30.65		49.19		77.42
		41.19		28.10		25.75		13.68		10.77		12.28		15.45		20.95		30.56		50.80		78.05	

**Table 7:** Recognition Rate of ProbeCL.

	GaitGAN	Probe Set View (Walking Wearing a Coat)																					
Ours		0°		18°		36°		54°		72°		90°		108°		126°		144°		162°		180°	
**Gallery set view**	0°		25.81		16.13		15.32		12.10		6.45		6.45		9.68		7.26		12.10		11.29		15.32
		25.06		17.08		16.75		12.09		7.52		7.00		10.12		8.56		15.12		10.45		14.29	
	18°		17.74		37.90		34.68		20.97		13.71		8.87		12.10		19.35		16.94		24.19		19.35
		16.52		36.50		35.16		22.85		14.75		8.69		12.04		18.64		16.48		23.66		20.78	
	36°		13.71		24.19		45.16		43.55		30.65		19.35		16.94		22.58		28.23		20.16		10.48
		14.55		24.06		45.52		44.45		30.42		20.48		17.84		22.49		29.06		19.74		10.28	
	54°		2.42		19.35		37.10		55.65		39.52		22.58		29.03		29.84		29.84		16.94		8.06
		4.60		20.89		36.22		54.90		40.64		23.90		29.15		28.70		28.52		16.50		8.72	
	72°		4.84		12.10		29.03		40.32		43.55		34.68		32.26		28.23		33.87		12.90		8.06
		5.87		14.76		28.67		39.87		44.70		35.40		33.65		28.19		32.94		11.50		7.08	
	90°		4.03		10.48		22.58		31.45		50.00		48.39		43.55		36.29		31.45		13.71		8.06
		3.06		7.52		15.67		23.74		45.68		47.57		40.76		31.69		24.54		10.70		5.50	
	108°		4.03		12.90		27.42		27.42		38.71		44.35		47.58		38.71		32.26		15.32		4.84
		4.15		11.50		26.49		27.08		38.97		45.00		48.65		39.07		33.00		15.60		4.55	
	126°		10.48		10.48		23.39		27.42		26.61		25.81		37.10		45.97		41.13		15.32		10.48
		10.50		9.97		22.45		27.69		25.98		25.08		37.25		99.7		41.50		15.30		9.63	
	144°		8.87		13.71		26.61		22.58		18.55		19.35		21.77		35.48		43.55		20.97		12.90
		8.80		14.55		25.97		23.06		19.29		19.26		20.47		35.04		42.98		21.59		12.70	
	162°		14.52		18.55		20.97		17.74		12.10		12.10		17.74		21.77		37.10		35.48		21.77
		15.00		18.98		20.65		18.06		11.97		10.55		15.64		20.08		37.68		35.50		22.86	
	180°		17.74		13.71		11.29		6.45		10.48		5.65		6.45		5.65		14.52		29.03		27.42
		16.57		13.67		10.84		6.71		10.09		5.76		6.32		6.79		13.87		27.03		28.84	

**Table 8:** Rank-1 accuracy obtained using different feature fusion modules.

Rank-1 Accuracy				
View	Concatenation	SENet	FPN	Ours
0°	60.8	79.3	75.6	80.5
90°	66.5	88.5	80.0	88.6
135°	65.6	85.0	77.9	86.4

**Table 9:** Rank-1 and Rank-5 accuracy obtained by testing data collected under real scenarios.

View	Rank-1 Accuracy	Rank-5 Accuracy
0°	80.5	88.7
90°	88.6	95.2
135°	86.4	90.6
Average	85.2	91.5

**Table 10:** Test results of short-time video input in real scenarios.

Stage	Silhouette Analysis			Keypoint Analysis			Recognition		
No.	1	2	3	1	2	3	1	2	3
Time (s)	32.06	40.02	36.75	7.65	6.62	6.56	5.33	5.42	5.66

## Data Availability

The OUMVLP-Pose is available at http://www.am.sanken.osaka-u.ac.jp/BiometricDB/GaitLPPose.html accessed on 21 May 2023 and CASIA-B is available at http://www.cbsr.ia.ac.cn/china/Gait%20Databases%20CH.asp accessed on 21 May 2023. For privacy reasons, please contact the corresponding author for requests to use test data in real scenarios.
